# Comparisons of Resistance Training and “Cardio” Exercise Modalities as Countermeasures to Microgravity-Induced Physical Deconditioning: New Perspectives and Lessons Learned From Terrestrial Studies

**DOI:** 10.3389/fphys.2019.01150

**Published:** 2019-09-10

**Authors:** James Steele, Patroklos Androulakis-Korakakis, Craig Perrin, James Peter Fisher, Paulo Gentil, Christopher Scott, André Rosenberger

**Affiliations:** ^1^School of Sport, Health, and Social Sciences, Solent University, Southampton, United Kingdom; ^2^Ukactive Research Institute, London, United Kingdom; ^3^Faculty of Physical Education and Dance, Federal University of Goias, Goiânia, Brazil; ^4^Department of Exercise, Health, and Sport Sciences, University of Southern Maine, Portland, ME, United States; ^5^Space Medicine Team, ISS Operations and Astronaut Group, Directorate of Human and Robotic Exploration Programmes, European Astronaut Centre, Cologne, Germany

**Keywords:** resistance training, cardio, aerobic training, microgravity, space

## Abstract

Prolonged periods in microgravity (μG) environments result in deconditioning of numerous physiological systems, particularly muscle at molecular, single fiber, and whole muscle levels. This deconditioning leads to loss of strength and cardiorespiratory fitness. Loading muscle produces mechanical tension with resultant mechanotransduction initiating molecular signaling that stimulates adaptations in muscle. Exercise can reverse deconditioning resultant from phases of detraining, de-loading, or immobilization. On Earth, applications of loading using exercise models are common, as well as in μG settings as countermeasures to deconditioning. The primary modalities include, but are not limited to, aerobic training (or “cardio”) and resistance training, and have historically been dichotomized; the former primarily thought to improve cardiorespiratory fitness, and the latter primarily improving strength and muscle size. However, recent work questions this dichotomy, suggesting adaptations to loading through exercise are affected by intensity of effort independent of modality. Furthermore, similar adaptations may occur where sufficient intensity of effort is used. Traditional countermeasures for μG-induced deconditioning have focused upon engineering-based solutions to enable application of traditional models of exercise. Yet, contemporary developments in understanding of the applications, and subsequent adaptations, to exercise induced muscular loading in terrestrial settings have advanced such in recent years that it may be appropriate to revisit the evidence to inform how exercise can used in μG. With the planned decommissioning of the International Space Station as early as 2024 and future goals of manned moon and Mars missions, efficiency of resources must be prioritized. Engineering-based solutions to apply exercise modalities inevitably present issues relating to devices mass, size, energy use, heat production, and ultimately cost. It is necessary to identify exercise countermeasures to combat deconditioning while limiting these issues. As such, this brief narrative review considers recent developments in our understanding of skeletal muscle adaptation to loading through exercise from studies conducted in terrestrial settings, and their applications in μG environments. We consider the role of intensity of effort, comparisons of exercise modalities, the need for concurrent exercise approaches, and other issues often not considered in terrestrial exercise studies but are of concern in μG environments (i.e., O_2_ consumption, CO_2_ production, and energy costs of exercise).

## Introduction

The physiological responses and adaptations to prolonged periods spent in microgravity (μG) environments have been described as a “classical” topic within the field of environmental and applied exercise physiology (Grassi, 2018). Indeed, the resultant deconditioning of numerous physiological systems and loss of strength, power, and cardiorespiratory fitness is well documented (Tesch et al., 2005; Lang et al., 2006; Trappe et al., 2009; Moore et al., 2010; Platts et al., 2014; Bloomberg et al., 2015). Exercise has long been used as the primary countermeasure for μG-induced deconditioning and the history of this has been detailed in an accompanying paper introducing this Research Topic (Scott et al., 2018). Recent reviews (Loenneke et al., 2012; Carpinelli, 2014) have also discussed the considerable attempts to solve the issue of how best to employ this countermeasure in μG environments. Many of these attempts revolve around what could be considered “engineering-based” solutions to employ the traditional exercise modalities often used on Earth as countermeasures for similar deconditioning (e.g., detraining, de-loading, disease, or immobilization). Broadly speaking, exercise is often (though not exclusively) dichotomized into two primary modalities, aerobic training (or “cardio”) and resistance training, with the former primarily thought to stimulate improvements in cardiorespiratory fitness, and the latter thought primarily to stimulate improvements in strength, power, and muscle size. Approaches to solve the issue of performing these typical modalities in μG have included deployment of currently used equipment on the International Space Station (ISS). For example, the Combined Operational Load Bearing External Resistance Treadmill (COLBERT)/Treadmill 2 (T2), Cycle Ergometer with Vibration Isolation and Stabilization System (CEVIS), and Advanced Resistive Exercise Device (ARED) among others such as suits for muscle loading (Penguin-3), lower body negative pressure (Chibis), and lower body g-loading (Kentavr), in addition to electrical stimulators (Tonus-3). The different agencies involved have to date employed a range of countermeasure protocols using such devices (Loehr et al., 2015; Yarmanova et al., 2015; Petersen et al., 2016). However, with the planned decommissioning of the ISS in 2024 (the earliest point in time though it may continue past this date), and future goals of manned moon and Mars missions, “engineering-based” solutions to apply both traditional “cardio” and resistance training exercise modalities inevitably present issues. These primarily relate to the mass, size, energy use, heat production, and ultimately cost of devices. It is therefore necessary to identify exercise countermeasures to combat the losses in strength, power, muscle mass, and cardiorespiratory fitness while limiting these issues.

However, this is not a simple task. Research on astronauts actually experiencing μG, particularly over extended periods of time, presents a number of barriers including logistics such as device sizes, costs, and participant sample sizes due to both difficulty in recruiting and the considerable between participant variability in many outcome measures often of interest. As such, “analogs” for μG environments are often used, with the most common being the bed rest study. A recent issue of *Medicine and Science in Sports and Exercise* detailed the results of NASA’s relatively recent 70-day bed rest study. This involved the use of concurrent resistance training and “cardio” exercise, in addition to testosterone supplementation, as countermeasures to deconditioning in a range of physiological systems (Cromwell et al., 2018; Dillon et al., 2018; Mulavara et al., 2018; Murach et al., 2018; Ploutz-Synder et al., 2018; Scott et al., 2018). The exercise interventions examined in the aforementioned bed rest study have been somewhat influenced by the recent body of literature supporting the use of high effort interval based “cardio” protocols [i.e., High Intensity Interval Training (HIIT)] as this was implemented as part of the concurrent program. The SPRINT protocol was designed to require less time and be performed at high intensities of effort including: HIIT performed using a custom-built vertical treadmill; resistance training using a custom built horizontal squat device; and both HIIT and resistance training using a flywheel device. Further, work from Russia has historically detailed the countermeasures used with cosmonauts (Kozlovskaya et al., 1995, 2015; Kozlovskaya and Grigoriev, 2004) and recently has compared the effects of either treadmill training with alternating sessions of higher and lower effort HIIT (*n* = 7), or traditional continuous endurance treadmill training (*n* = 8; Fomina et al., 2016). They examined the cosmonauts over 189 ± 12.4 days aboard the ISS and found the HIIT protocol to result in maintenance of most pre-flight outcomes compared to the losses seen in the traditional endurance training. The Canadian Space Agency (CSA), Japan Aerospace Exploration Agency (JAXA), and European Space Agency (ESA) are currently using similar protocols that have been detailed extensively (Loehr et al., 2015).

It seems that developments within terrestrial studies in exercise physiology have been incorporated into the research programs of those μG analogs. However, other recent work based upon terrestrial studies (Fisher and Steele, 2014) has also begun to question the traditional dichotomy regarding resistance training and “cardio” exercise, including the need for concurrent training approaches. Furthermore, the same authors suggest that adaptations to loading through exercise may be primarily influenced by the intensity of effort employed independent of modality, and that similar adaptations might be achieved with differing exercise modes assuming sufficient intensity of effort is reached. Indeed, this questioning is of interest to pursue since it could imply that a lower volume of overall exercise might be adequate for astronaut’s physical fitness training in μG. Though astronauts value their time for exercise for wider personal wellbeing and many would likely prefer more time for such activity, from an operational perspective any approach that might help reduce time spent exercising could be considered beneficial, particularly if it can also yield similar physiological outcomes compared with greater volumes of exercise.

Contemporary developments in understanding of the applications of, and subsequent adaptations to, exercise-induced muscular loading in terrestrial settings have advanced in recent years. It may be appropriate to revisit the evidence to better understand how exercise might be applied in μG for potential investigation in future studies of μG analogs such as bed rest studies. In this brief review, we focus on the application of resistance training and “cardio” training modalities. Topics covered include: the role of intensity of effort, comparisons of exercise modalities (resistance training vs. “cardio”), and whether there is a need for concurrent exercise approaches currently used as countermeasures, as well as other issues often not considered in terrestrial exercise studies but which are of concern in μG environments such as O_2_ consumption and CO_2_ production in addition to energy costs of exercise. We note that resistance training and “cardio” training neither reflect the entirety of possible approaches to exercise countermeasures, nor are cardiorespiratory fitness, strength, power, and muscle size, the only outcomes that might be of interest when discussing the deconditioning that occurs in response to μG environments. Scott et al. (2019) list a number of alternative countermeasure approaches in addition to other outcomes of interest in their Introduction to this Research Topic. We encourage the reader to consider the other reviews covered in this Research Topic, which discuss many of these alternative approaches. Further, we add that the recent advances in understanding of exercise response from terrestrial studies presented here should be considered as candidates for further research within μG analogs. Their ability to be effectively implemented into true μG settings not be assumed based upon terrestrial studies.

## Intensity of Effort: A Possible Equalizer for Adaptation?

The intensity of effort during exercise can be defined in relation to the current ability to meet the demands of the task being attempted, and for resistance training this is often considered with respect to the proximity to momentary failure (Steele, 2014; Steele et al., 2017). The perception of that effort is thought to arise from the central motor command required to drive the musculature to perform the task being attempted (Marcora, 2009; Pageaux, 2016), and of course, this drive is influenced by the ability of that musculature to meet those demands, which can be determined by various fatigue processes and afferent feedback from the muscles (Steele and Fisher, 2018). Thus, it is thought that effort, both that required and perceived, is likely intrinsically linked to motor command and motor unit recruitment (de Morree et al., 2012; Guo et al., 2017; Potvin and Fuglevand, 2017).

As noted, it has recently been speculated that, assuming effort is matched, adaptations to exercise are likely to be similar (Fisher and Steele, 2014). Indeed, with respect to resistance training this appears to be the case for muscular adaptations. When performed to momentary failure, recent work suggests there may be little effect of load (Schoenfeld et al., 2017), repetition duration (Schoenfeld et al., 2015; Hackett et al., 2018; Carlson et al., 2019), muscle action (Fisher et al., 2016b), or whether “advanced” techniques are employed such as pre-exhaustion (Fisher et al., 2014), breakdown sets (Fisher et al., 2016a), or blood flow restriction (Barcelos et al., 2015; Farup et al., 2015). This is not to say that it is a requirement to train to a maximal intensity of effort (i.e., to momentary failure) to produce adaptation, or that doing so is necessarily optimal; indeed, findings regarding this are conflicting (Davies et al., 2016). Recently, it has also been shown that during high effort but non-momentary failure training there are similar adaptations irrespective of load and that these may even be similar to when training to momentary failure (Nóbrega et al., 2018). It is not clear what the dose–response nature of proximity to failure and thus effort is, or whether a threshold phenomenon might exist to optimize adaptation (Steele et al., 2017). However, what does seem clear is that when effort is high and appropriately matched, various resistance training manipulations yield very similar adaptations as noted by Phillips and Winett (2010): “…*effort is internal to the person, can be created with a variety of protocols, and is not dependent upon a specific amount of external force*”. As such, effort could be considered in both resistance training and “cardio” training as being determined primarily with respect to proximity to momentary failure.

The importance of high or maximal intensity of effort has become apparent and studies of concurrent exercise in μG simulations have begun to use these approaches for both “cardio” and resistance training (Cotter et al., 2015). However, it is not known whether similar effects are seen across modalities when either modality alone is performed. As an example, resistance training has been evidenced to result in improvements in cardiorespiratory fitness (Steele et al., 2012; Ozaki et al., 2013; Ashton et al., 2018), and “cardio” training to improve strength and muscle size (Konopka and Harber, 2014; Ozaki et al., 2015) though their comparative effects are less clear. Studies have attempted to compare “cardio” and resistance training modalities, some of which have appropriately controlled for effort, and duration. Others have examined what could be considered more traditional representations of the two approaches. In the following section, we will review these studies and consider whether the stimulus and adaptation resulting from exercise-induced loading is influenced by the modality used.

## Comparisons of Traditional “Cardio” and Resistance Training Approaches

Traditional approaches to “cardio” are often performed using locomotive or ergometer tasks (e.g., walking, jogging, running, cycling, rowing, etc.) in a continuous fashion with respect to duration at submaximal intensities of effort commonly determined relative to either maximal heart rate, heart rate reserve, VO_2_max, or sometimes using ratings of perceived effort scales. Sometimes they are performed using “high intensity interval approaches” though many studies that compare modalities still use submaximal intensities of effort and are unmatched compared to the resistance training approaches examined. Contrastingly, resistance training is often performed with external resistance of varying degrees relative to maximal strength provided by either free weights, machines, bodyweight, or some other implements (e.g., resistance bands), either with single or multiple sets of repetitions which may or may not be performed to momentary failure (but are often performed to a relatively high effort). Most people would recognize these approaches as being “ecologically valid” implementations of either “cardio” or resistance training (i.e., reflective or their implementation in “real-life” settings) and numerous studies have compared these forms.

When comparing resistance training and “cardio” approaches, though some studies suggest no significant differences for changes in cardiorespiratory fitness (Messier and Dill, 1985; Hepple et al., 1997; Sawczyn et al., 2015), the majority suggest that “cardio” type approaches favor cardiorespiratory fitness increases (Goldberg et al., 1994; Poehlman et al., 2000; Ferrara et al., 2006; Sillanpaa et al., 2008; Wilkinson et al., 2008; Ahtiainen et al., 2009). Similarly, for strength changes, though there are exceptions (Messier and Dill, 1985), the majority of research suggests that resistance training produces greater increases in strength than “cardio” type training (Goldberg et al., 1994; Poehlman et al., 2000; Ferrara et al., 2006; Sillanpaa et al., 2008; Wilkinson et al., 2008; Ahtiainen et al., 2009). Furthermore, a recent meta-analysis has also shown that resistance training produces more favorable changes in muscle hypertrophy compared to “cardio” type approaches (Grgic et al., 2018).

This body of research suggests a specificity of training response with respect to “cardio” and resistance training, with the former favoring cardiorespiratory fitness and the latter favoring strength and hypertrophy. However, these results apply to broad comparisons of these two ecologically valid approaches to training. Yet the results of the aforementioned comparative studies do not necessarily imply that the modality, and thus mechanical resistance (e.g., the load on a resistance exercise, or the power output on a cycle ergometer) itself independent of the manner in which exercise is performed with it, is influential with respect to adaptations. Many of the studies cited have often tested their outcomes in manners that might favor particular interventions. For example, cardiorespiratory fitness being tested on the modality for which the “cardio” intervention was trained, and conversely strength being tested as a one repetition maximum in the exercise for which the resistance training intervention was specifically trained. Further, as noted, in none of the aforementioned comparative studies were attempts made to control for the effort and duration of the two interventions. However, in recent years, there have been attempts to conduct research comparing across exercise modalities while controlling for effort and duration examining both the acute physiological responses in addition to the chronic physiological adaptations.

## Effort and Duration Matched Modality Comparisons

A number of studies have examined the influence of modality upon acute responses, typically focusing upon measures, which may be speculated to have a potential role in mediating chronic adaptations. Vilaça-Alves et al. (2016) compared both upper and lower body “cardio” (upper- and lower-body cycle ergometry) and resistance exercise (smith machine bench press and smith machine half squat) modalities during low intensity of effort with physiologically matched tasks (demands eliciting 4 mmol.L^−1^ of blood lactate). They examined the oxygen uptake responses between the two modalities finding no differences and concluded that the manner of exercise performance, and not the modality, was likely the primary determinant of this physiological response. More recently, Steele et al. (2018) compared the acute response of lower body “cardio” (recumbent cycle ergometry) to resistance exercise (leg press) during high intensity of effort tasks, matched for effort and duration (4 × 60 s sprints for “cardio” and 4 × 12 repetition maximum for resistance exercise with time matched using a 2 s concentric and 3 s eccentric repetition duration). They considered a range of physiological responses including oxygen consumption, respiratory exchange ratio, blood lactate, estimated energy expenditure, muscle swelling, and electromyography finding no differences between the modalities for any outcome. Steele et al. (2018) examined only amplitude based electromyographical variables but Noble et al. (2019) have also examined normalized (to % max) electromyographic amplitudes, which appear to be greater during typical resistance training (single leg knee extension) compared with “cardio” mode (single leg cycle ergometry) exercise performed to volitional failure. Unlike Steele et al. (2018), time to task failure in Noble et al. (2019) was unclear and thus, it is unknown if it was similar between conditions. Amplitude-based analyses may not reflect the entirety of motor units recruited where task durations differ, particularly if differing recruitment patterns are occurring (i.e., sequential recruitment of low to high threshold during low force tasks, and simultaneous recruitment of both low and high threshold motor units during high force tasks; Enoka and Duchateau, 2015; Fisher et al., 2017; Potvin and Fuglevand, 2017; Vigotsky et al., 2017). However, Kuznetsov et al. (2011) examined resistance training (knee extension) and “cardio” exercise (cycling) modalities performed to momentary failure (thus controlling for effort) using frequency based electromyographic analyses and reported that similar recruitment of motor units may occur during both modalities. These findings might be expected considering the possible link between effort and central motor drive as noted above. For example, motor unit recruitment for active muscles might be relative to task demands independent of the exercise modality, which might also be true for other physiological responses such as oxygen consumption, blood lactate production, muscle edema, etc.

Similarity in acute responses between modalities when effort and duration are matched has led to the hypothesis that the accompanying chronic physiological adaptations may therefore be similar as well (Fisher and Steele, 2014). However, to date, research examining this is limited. Only two studies have been published to our knowledge (Álvarez et al., 2017; Androulakis-Korakakis et al., 2018); though, our lab and others have been conducting research in this area, the initial findings of three further studies are also presented below.

Androulakis-Korakakis et al. (2018) examined an 8 week intervention of additional (i.e., alongside their normal training) “high intensity interval training” performed using either a “cardio” exercise modality (cycle ergometry) or a resistance training modality (squats and deadlifts) in powerlifting and strongman athletes. Both were performed 2x/week for 7 sets of either 30 s on the cycle ergometer or sets of ~16–30 s alternating with squats and deadlifts at a rating of perceived effort of 8–9 (on a 0–10 scale) and with 90 s rest between sets. Predicted VO_2_max using the step test and predicted 1 repetition maximum on the knee extension were selected as outcomes to avoid issues of specificity (as discussed above) of training modality affecting test outcomes. Both outcomes improved, yet there were no statistically significant differences between groups^1^ for change in predicted VO_2_max [Δ = 4.6 ml.kg.min^−1^ (95%CIs = 3.0 to 6.3) vs. Δ = 3.4 ml.kg.min^−1^ (95%CIs = 1.7 to 5.1) for “cardio” and resistance training, respectively; *p* = 0.259] or for change in predicted knee extension 1 repetition maximum [Δ = 7.1 kg (95%CIs = 4.4 to 9.7) vs. Δ = 6.9 kg (95%CIs = 4.2 to 9.5) for “cardio” and resistance training, respectively; *p* = 0.895]. It is surprising to see improvements in an already well-trained population such as this, and thus, it might be expected that results may translate to untrained populations as well.

Álvarez et al. (2017) recently compared 12 weeks of effort and duration matched “cardio” (cycle ergometer “high intensity interval training”) and resistance training (full body resistance training including biceps curls, knee extension, shoulder press, and upright rows) 3x/week in insulin resistant women. Each was performed with the same work:rest intervals (60 s:120 s) for 12 sets at a rating of perceived effort of 8–10 (on a 0–10 scale). Their outcomes included body composition/anthropometry, cardiovascular outcomes, plasmatic concentrations, strength, and endurance. For most of these, they found improvements but no statistically significant differences between groups. However, for strength there were greater changes in the resistance training group, though strength was tested using 1RM on the same exercises used in training (measured as 1RM in biceps curl, knee extension, shoulder press, and upright row). To the contrary, endurance performance was tested as 2 km walking test which improved in both groups with no statistically significant differences between them (both improving by ~2 min; *p* = 0.284).

More recently, Gil-Sotomayor et al. (2018) presented results from a study comparing 12 weeks of “high intensity interval training” combined with a low carbohydrate high fat diet using either “cardio” (cycle and/or treadmill ergometer) or resistance training (pull-down, leg press, bench press, dumbbell row, sumo squat, and push-ups) 3x/week. Both were effort and duration matched and performed with the same work:rest ratio (60 s:60 s) for 10 sets each with a target rating of perceived effort of 16–18 (on a 6–20 scale). They reported that VO_2_ peak, body mass, fat mass, and visceral fat were all significantly improved in both groups. For VO_2_ peak, Hedge’s *g* for changes were 0.57 and 0.53 for “cardio” and resistance training, respectively. Of note, lean body mass did decrease slightly for the “cardio” modality (*g* = −0.07) and did not change for resistance training (*g* = 0.00).

**Figure 1:**
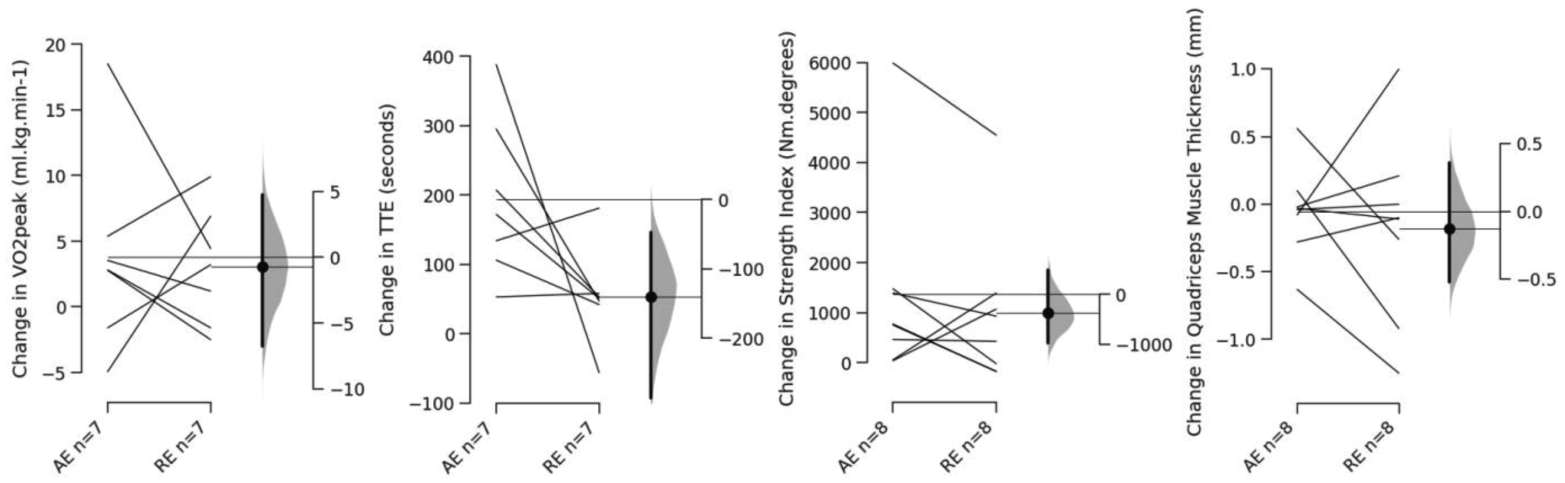
From left to right, individual responses and paired comparisons (floating axis showing mean difference with 95% confidence intervals) for change in VO_2_ peak, time to exhaustion during progressive ramp unilateral cycle test, isometric knee extension strength index (area under torque curve), and quadriceps muscle thickness; AE, Aerobic Exercise/“Cardio” Group; RE, Resistance Training Group.

Finally, our labs are currently completing a training intervention study using a similar approach to the aforementioned acute responses study by Steele et al. (2018) using a within-participant design, in addition to having recently completed a further between-group training intervention study. In this within-participant study, Armes et al. (unpublished) have begun examining lower body “cardio” (unilateral recumbent cycle ergometry) with lower body resistance exercise (unilateral leg press) matched for effort and duration (4 × 30 s sprints and 4 × ~5–7 repetitions to momentary failure at 2 s:3 s repetition duration for “cardio” and resistance exercise, respectively). A within-participant design, whereby participants limbs were randomized to conditions in a counterbalanced fashion based upon dominant limb, has been used to increase power and precision for estimates by reducing between-condition variation from independent samples (MacInnis et al., 2017). The training consists of two sessions per week for 8 weeks with both conditions performed in each session with the order alternated in order to avoid any specific order effects of performing one condition prior to the other. Outcomes being examined include unilateral VO_2_ peak and time to exhaustion during an incremental exercise test on an upright cycle ergometer, unilateral isometric knee extension strength, and ultrasound measured quadriceps muscle thickness. Based on precision (i.e., desired width of confidence intervals set at 0.5 population standard deviation), target *N* was calculated at 21, though here we present preliminary data for *N* = 8 participants^2^ (Figure 1). Despite the within-participant design, there is evidently considerable variation or noise in the data. However, at this stage, descriptively it seems that VO_2_ peak improves slightly on average with only a small difference between conditions for this change and the same seems to be the case for strength. Time to exhaustion, however, though improving for both conditions has clearly increased to a greater degree in the “cardio” condition. Although training was performed with a recumbent cycle ergometer and testing was on an upright cycle ergometer, there was sufficient transfer with respect to the specificity of motor tasks that improved time to exhaustion independently of VO_2_ peak. Lastly, and likely reflective of the inherent noise in the measures, or individual variation in response, quadriceps muscle thickness did not clearly change for either condition and the average changes seen so far fall within the technical error of measurement for our lab (0.14 cm).

A between-group training intervention study completed by Silva et al. (2019) was conducted in trained males (with a minimum of 6 months prior resistance training experience) and using either resistance training on a leg press (4 × ~10–12 repetitions to momentary failure at concentric to eccentric 1 s:2 s repetition duration ratio) or “cardio” training on an upright cycle ergometer (4 × 30 s sprints). Outcomes included strength measured using knee extension 10RM, VO_2_peak measured using a maximal incremental treadmill protocol, and body composition measures of the legs using dual X-ray densitometry (DXA). Analysis using ANCOVA with baseline values as covariates revealed no statistically significant between group differences (*F*_(1,22)_ = 0.261, *p* = 0.614) for change in strength with improvements in both the leg press group [estimated marginal mean (95% CIs) Δ = 10.1 kg (6.9 to 13.3)] and cycle ergometer group [estimated marginal mean (95% CIs) Δ = 9.1 kg (6.1 to 12.2)]. There were also no statistically significant between-group differences for changes in total leg mass (*F*_(1,22)_ = 1.589, *p* = 0.221), leg lean mass (*F*_(1,22)_ = 0.491, *p* = 0.491), or leg fat mass (*F*_(1,22)_ = 1.238, *p* = 0.278) and changes within-groups were negligible. For changes in VO_2_peak, however, there was a statistically significant between-group difference (*F*_(1,22)_ = 5.926, *p* = 0.023) revealing greater increase for the cycle ergometer group [estimated marginal mean (95% CIs) Δ = 5.66 ml.kg.min^−1^ (2.63 to 8.68)] compared with the leg press group [estimated marginal mean (95% CIs) Δ = 1.23 ml.kg.min^−1^ (−1.92 to 4.38)]. Considering the different training and testing modality, this change is interesting and contradicts results reported by others regarding changes in cardiorespiratory fitness measured with a range of tests and using effort matched protocols (Álvarez et al., 2017; Gil-Sotomayor et al., 2018), including those in trained populations (Androulakis-Korakakis et al., 2018). However, the study by Silva et al. (2019) there was considerable variation in maximal criteria from the incremental treadmill protocol with none of the participants reaching a respiratory exchange ratio >1.15 and also a number of participants showing differences in end of test max heart rate (>10 beats.min^−1^) between pre- and post-tests suggesting that truly maximal efforts may not have occurred. In contrast, all participants that have been tested so far by Armes et al. (unpublished) have met maximal criteria for the incremental exercise test (RER >1.15, hear rate ± 10 beats.min^−1^ of age predicted max, Borg scale rating of 20, and blood lactate >8 mmol.L^−1^).

**Figure 2:**
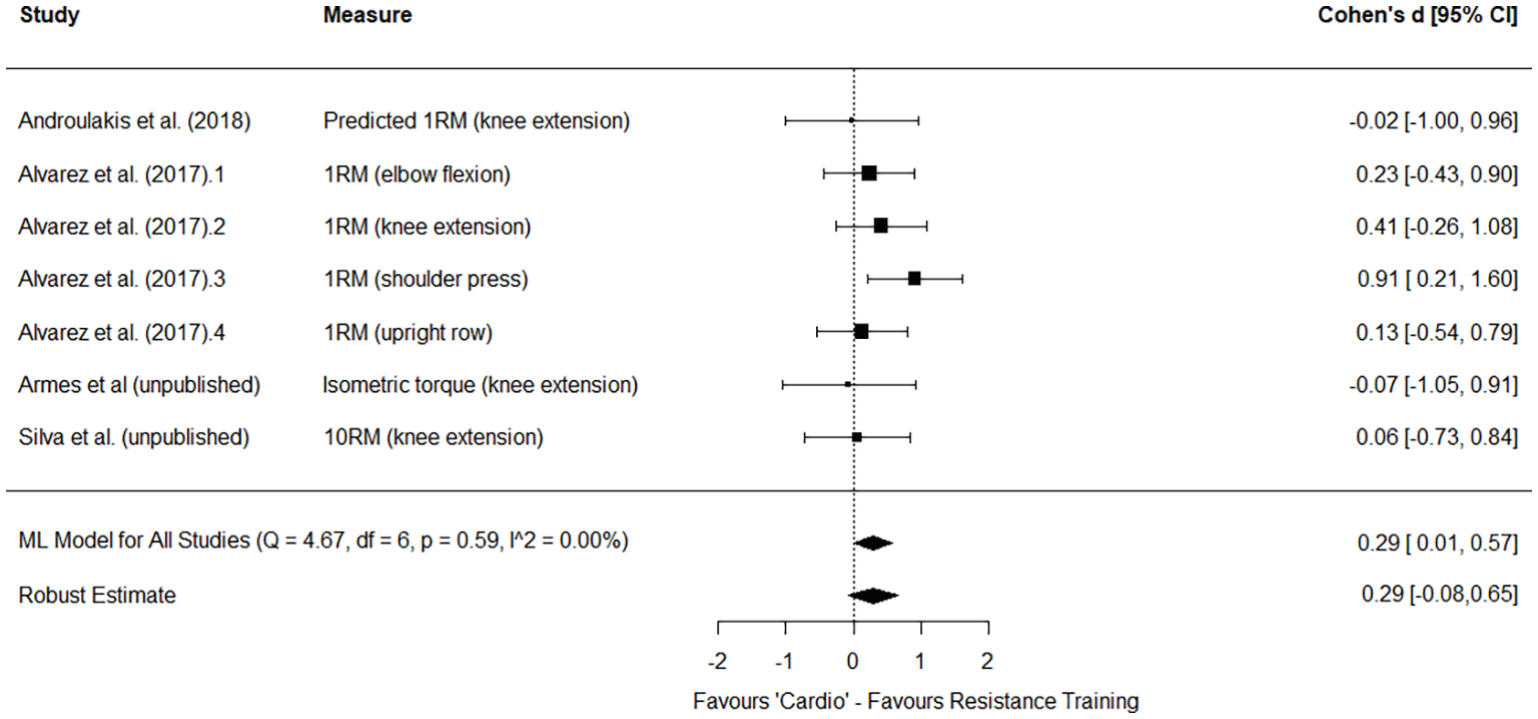
Forest plot for multilevel (ML) mixed effects meta-analysis of strength outcomes from studies using effort matched designs to compare “cardio” and resistance training modalities.

**Figure 3:**
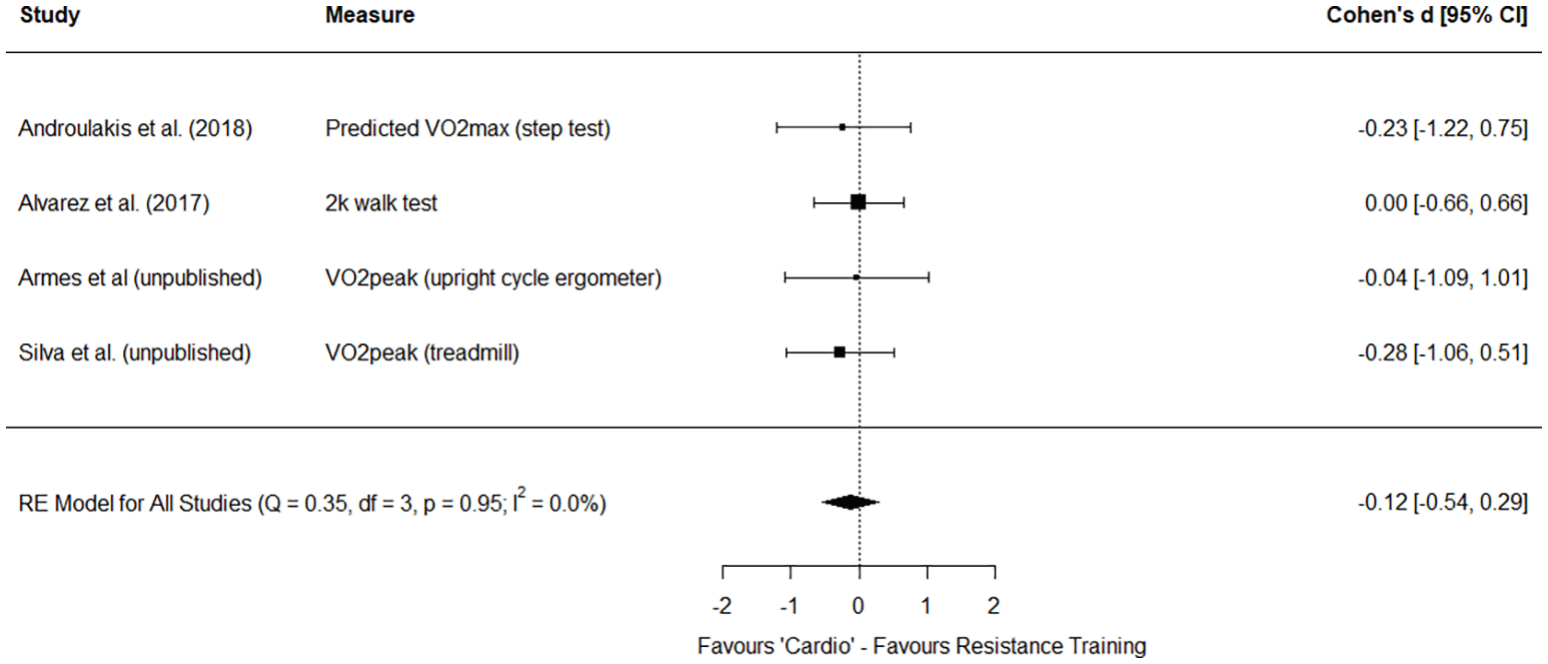
Forest plot for random effects (RE) meta-analysis of cardiorespiratory fitness outcomes from studies using effort matched designs to compare “cardio” and resistance training modalities.

Considering the pattern of findings from this emerging body of research, there does seem to be evidence supporting the hypothesis of physiological adaptations and responses being primarily determined by effort, and less influenced by modality. In fact, random effects meta-analysis^3^ comparing effect sizes between “cardio” and resistance training modalities for 4 of the studies discussed above (Álvarez et al., 2017; Androulakis-Korakakis et al., 2018; Silva et al., 2019; Armes et al., unpublished) seem to support that presently there is little evidence suggesting a difference between modalities when effort is controlled for. For strength, the effect size favored resistance training with a small effect (though this was not significant with the robust estimate) though with moderate precision for the estimate (Figure 2) and trivially for changes in cardiorespiratory fitness measures in “cardio” training (Figure 3). This is an emerging area and despite the similarity of findings across studies there is clearly further work required to approach a more precise understanding of the differences, or lack thereof, in adaptations produced by different yet effort matched modalities. However, the potential implications of what we understand so far are discussed below. For now, we briefly look to other considerations often overlooked in research considering exercise countermeasures to μG-induced deconditioning.

## Other Considerations: O_2_ Consumption, CO_2_ Production, and Energy Costs

Factors which are often underappreciated when considering exercise countermeasures for μG-induced deconditioning are that, compared to rest, exercise increases O_2_ consumption, CO_2_ production, and energy costs. Combined, the former two mean that more O_2_ needs to be produced *via* electrolysis and additional CO_2_ need to be removed. Furthermore, the additional energy expenditure from exercise requires that additional resources are necessary to replenish that energy used by the astronauts (i.e., food rations). This is particularly noteworthy since data shows an increased energy requirement (1.4 × resting metabolic rate) through elevations in basal metabolic rate in both simulated (Acheson et al., 1995), and μG environments (Stein et al., 1999). As such, it is of interest to consider the effects of “cardio” and resistance training upon O_2_ consumption, CO_2_ production, and energy costs. However, there is little research specifically examining this area, especially that which has controlled for effort and duration of exercise to examine the effects of modality. Work matched comparisons of different resistance training approaches (circuit style or traditional consecutive sets) show that O_2_ consumption (aerobic energy expenditure during exercise and rest interval) and total energy expenditure is similar (Aniceto et al., 2013). Although, it is noteworthy that anaerobic energy expenditure estimated from production of blood lactate was higher in consecutive sets compared with circuit style of resistance training suggesting CO_2_ production may be higher (Aniceto et al., 2013). Thus, other technical elements of how resistance training protocols are manipulated may be important to consider. For example, consecutive sets of resistance training when performed not to momentary failure likely results in increasing effort with each set due to residual fatigue. Though it would seem that effort may be an important factor impacting the similarity of responses and adaptations to different exercise modalities. Scott and Earnest (2011) have shown that aerobic, anaerobic, and recovery energy expenditures are all higher with resistance training performed to momentary failure even when work is matched. As noted earlier, it appears that O_2_ consumption, CO_2_ production, and energy costs are more a function of the intensity of effort of exercise as opposed to the modality.

## Discussion and Suggestions for Potential Research and Solutions

Though currently there is limited research examining the role of modality of exercise, what has been conducted is suggestive of greater similarity in the physiological responses and adaptations between “cardio” and resistance training than historical dichotomies would predict, assuming effort and duration are matched. Considering this, the prevailing use of concurrent training modalities on the ISS to counter μG-induced deconditioning of physiological systems may be unnecessary. Indeed, of interest is that similar changes can occur even in well-trained participants as reported by Androulakis-Korakakis et al. (2018) and found by Silva et al. (2019) despite the fact that the adaptive response to exercise is attenuated in trained persons. Astronauts undergo considerable physical preparation prior to entering μG environments and then subsequently experience deconditioning akin to that which might occur from a period of detraining. Periods of detraining have been shown to restore sensitivity of anabolic signaling pathways in skeletal muscle (Ogasawara et al., 2013). Thus, considering the similar responses reported in both trained (Androulakis-Korakakis et al., 2018; Silva et al., 2019) and also untrained populations (Álvarez et al., 2017; Gil-Sotomayor et al., 2018) it seems reasonable to speculate that single modality approaches to counter μG-induced deconditioning might be appropriate. This could potentially halve the time required by astronauts for engagement in training as a countermeasure and potentially have concurrent impact on reducing volume of space taken up by equipment, as well as reducing O_2_ consumption, CO_2_ production and energy use by the astronauts in addition to heat production and energy use from exercise devices themselves.

In this sense, it could also be argued that either “cardio” or resistance training approaches might therefore be chosen as the preferred modality, though we would argue that resistance training approaches in general include further benefits over and above “cardio” based approaches which we discuss below. As noted, the choice of a single modality may solve issues of time, volume of space, and energy use/heat production. Moreover, resistance training in contrast to “cardio” approaches can be performed in a manner that better addresses these issues. For example, isometric resistance training has been shown to require lower VO_2_ consumption and energy cost compared with dynamic forms (Scott et al., 2015). It can also be performed without the need for external devices (“just” proper fixation in the spacecraft is needed in most exercises). Isometric resistance training can also be performed using contralateral limb provided resistance (i.e., using one limb to resist the movement of the other) which can elicit similar electromyographical activity as traditional free weight training (Fisher et al., 2016c). Maximal isometric co-contraction approaches have also been examined and found to be effective for upper limb (Maeo et al., 2014a,b) and trunk and hip musculature (Tayashiki et al., 2016), though may be potentially less effective for the lower limbs (Maeo and Kaneshisa, 2014). There may be some concerns with the use of isometrics for countering μG deconditioning. Haddad et al. (2006) suggested that their use did not counteract the downregulation of anabolic signaling that occurred from short term unloading, though follow up research from their lab found that, assuming sufficient volume of exercise is performed, signaling is similar independent of muscle action (Garma et al., 2007). This being said, early work has suggested that adaptations to contractile kinetics may differ between isometric and concentric resistance training (Duchateau and Hainaut, 1984) and so perhaps a combination of the two may be best. Other examinations of “no load” resistance training have been reported showing that dynamic movement coupled with maximal voluntary effort to activate the muscle involved produces high electromyographical activity independent of training status, limb dominance, movement velocity, or the use of visual feedback (Gentil et al., 2017), and such training has been shown to produce similar increases in muscle size and strength compared to traditional dynamic free weight training (Counts et al., 2016). In addition, there are more simple approaches to providing external resistance for dynamic resistance training compared to those primarily used in current μG environments. For example, partner assisted manual resistance training has been shown to be similarly effective to more traditional approaches (Dorgo et al., 2009), however, at least for missions to the ISS where crew time is critical and their schedule is often constrained, this training regimen may be less favorable. Also, a simple self-powered rope trainer has been examined for possible use in μG environments and shown to be equally effective in increasing strength as traditional free weight resistance training (Behringer et al., 2016). Further, even when performed using minimal doses (twice a week using 3 multi-joint exercises performed for two sets to a rating of perceived effort of 8 out of 10, where 10 would be the point of momentary failure), elastic resistance bands have been shown to produce similar strength changes to traditional free weight or machine-based resistance training (Souza et al., 2019).

Thus, there may be the potential to employ resistance training based approaches alone and in such a way where the equipment requirements are further reduced. However, were future research to examine and validate resistance training as an effective single modality in μG analog studies, for it to be considered appropriate as the single modality approach for space missions, other aspects need also to be taken into consideration. We have focused here upon cardiorespiratory fitness, strength, and muscle size as they have been the primary outcomes for terrestrial comparative studies. However, specific performance levels for certain tasks would likely be needed to maintain the ability to perform these specific motor patterns. Without inclusion of at least some walking/running there may be a potential injury risk due to altered gait/running patterns, and vice versa, were only a single “cardio” modality approach used there may be problems in lifting/carrying objects on the surface of Mars. Of course, it should be noted that at present, research is limited and further evidence would likely be required to conclusively suggest a single modality as being sufficient. Further, the suggested approaches mentioned specifically for application of resistance training have not been examined in direct comparison to “cardio” based modalities. There is scope for this research to be conducted and we feel should be encouraged for future μG analogs such as bed rest studies. In most of the studies cited above though, maximal or near-maximal efforts were employed and in many cases novel resistance training techniques were compared to traditional free weight based resistance training. In combination with the similar positive results between different resistance training approaches when effort is matched, along with the emerging evidence suggesting “cardio” and resistance training modalities may produce similar adaptations if effort is matched, we pose the hypothesis that adaptations to exercise-induced loading may be more influenced by the manner in which they are performed, primarily the effort put forth, as opposed to the nature of the specific modality utilized. This may result from the similar physiological stimulus experienced when effort is matched. Indeed, as noted effort may be intrinsically linked to motor unit recruitment for performance of physical tasks. However, it should be noted that some work has suggested that, at least at very low efforts (i.e., postural support from m. soleus and m. gastrocnemius during “dry immersion” involving <7% of maximal voluntary force) motor unit recruitment order may be altered with μG exposure (Shiqueva et al., 2015). Whether this would be the case under higher effort exercise conditions in μG is less clear. As such, though we feel there is considerable scope for lessons learned from traditional terrestrial exercise science studies about effort-based paradigms for exercise-induced skeletal muscle loading to be applied to μG analogs and environments, there remains the need to examine the effects of them both acutely and chronically in these settings.

We reiterate here that resistance training and “cardio” training do not reflect the entirety of possible approaches to exercise countermeasures, nor are cardiorespiratory fitness, strength, power, and muscle size the only outcomes that might be of interest when discussing the deconditioning that occurs in response to μG environments. However, this review presents emerging evidence from terrestrial setting that, where effort and duration are matched, both resistance training and “cardio” training may produce largely similar physiological responses and adaptations. These recent advances in understanding of exercise response from terrestrial studies should be considered as candidates for further research within μG analogs and for a wider range of outcome measures as a step toward their consideration for implementation into true μG settings.

## Data Availability

The datasets generated for this study are available on request to the corresponding author.

## Author Contributions

JS and PG contributed to conception and design of the review. JS performed the statistical analysis and wrote the first draft of the manuscript. PA-K, CP, JF, PG, CS, and AR wrote sections of the manuscript. All authors contributed to manuscript revision, read and approved the submitted version.

### Conflict of Interest Statement

The authors declare that the research was conducted in the absence of any commercial or financial relationships that could be construed as a potential conflict of interest.

## Footnotes

^1^Note, data has been reanalyzed using JASP (version 0.9.1, University of Amsterdam, Netherlands) for this study with ANCOVA for the change in outcome (post-minus pre-scores) using pre-scores as covariates.

^2^Note, one participant did not complete post testing for VO_2_ peak and time to exhaustion due to illness and so *N* = 7 for these outcomes.

^3^Performed using the “metafor” package in R (version 3.5.1; R Core Development Team, https://www.r-project.org/) and an alpha of 0.05 considered in all tests. Between group effect sizes using Cohen’s *d* were calculated for differences between groups in change scores, and pooled change score standard deviations used as the denominator (Morris, 2007; Borenstein et al., 2009; Dankel and Loenneke, 2018). The study of Gil-Sotomayor et al. (2018) was not included due to data on variance being unavailable. Because of the 4 strength outcomes used in Álvarez et al. (2017) a multilevel mixed effects meta-analysis was performed for strength changes with cluster robust variance estimation. ES for *d* were interpreted with reference to Cohen’s (1988) thresholds; trivial (<0.2) small (0.2 to <0.5), moderate (0.5 to <0.8), and large (>0.8) and positive ES values indicated higher scores of the outcome in favor of the resistance training group.
